# The shape of a kiki: Sound symbolism affects production of figures

**DOI:** 10.3758/s13423-026-02864-0

**Published:** 2026-02-17

**Authors:** Padraic Monaghan, Simya Aravamuthan

**Affiliations:** Department of Psychology, Lancaster University, Lancaster, LA1 4YF UK

**Keywords:** Sound symbolism, Bouba-kiki, Drawings, Cross-modal effects

## Abstract

Previous laboratory-based studies of sound symbolism tend to use established sound and meaning distinctions and find strong effects in participants forming mappings between them. Yet sound symbolism in natural language is extant, but only a weak effect. Without constraints and cueing to categorical distinctions in meaning, are sound symbolic effects still observable? We asked 40 participants to draw figures in response to 20 novel words presented auditorially varying in consonant and vowel properties. Plosives and front vowels resulted in more angular and straight-lined figures, whereas sonorants and back vowels elicited more rounded and curved shapes. The results are consistent with the classic bouba-kiki (takete-maluma) effect and show that both consonants and vowels can systematically shape sound-meaning mappings in the absence of explicit constraints. While natural language statistics may partly explain these effects, the consistency across phonemes – including those not frequent in English – suggests deeper perceptual or cognitive origins.

**Supplementary Information:**

The online version contains supplementary material available at 10.3758/s13423-026-02864-0.

## Introduction

A preponderance of studies of sound symbolism have shown that words’ sounds can affect decisions about how those words match onto potential meanings (Sidhu & Pexman, 2017). Such sound symbolism effects provide insight into how wider cognitive and perceptual cross-modal correspondences may influence language processing and language structure (Dingemanse et al., 2015). A long-standing and influential sound symbolic effect is known as the *takete-maluma* (Köhler, 1929) or the kiki/bouba effect (Ramachandran & Hubbard, 2001), where words that vary in consonantal sonorance and voicing and vowel position drive implicit and explicit decisions, memory and learning about mapping onto angular or rounded shapes (Brand et al., 2018; Ćwiek et al., 2021; Imai et al., 2008; Kantartzis et al., 2011; Lockwood et al., 2016; Nygaard et al., 2009; Parise & Spence, 2012; Sonier et al., 2020; Westbury, 2004).

Both consonants and vowels have been shown to be involved in sound symbolic effects. In a systematic review, Lockwood and Dingemanse (2015) identified a set of sound symbolic properties that had been verified in (forced choice) tests of various sound-meaning mappings, spanning both voicing and sonorance of consonants, and height and position of vowels. For the angular-rounded distinction, consonant voicing (unvoiced are angular, voiced are rounded), sonorance (plosives are angular, sonorant are rounded), and vowel position (front vowels are angular, back vowels are rounded) were attested as category distinctions. In terms of their relative strength, consonant distinctions have tended to exert a greater effect than vowels in relating to angular and rounded shapes (Fort et al., 2015; Monaghan et al., 2012; Nielsen & Rendall, 2012; Ozturk et al., 2013).

However, what all these studies have in common is that the distinctions between sounds and between meanings are given within the study design: Words reflecting distinctions in the sounds tend to be paired with meanings which vary along one meaning dimension. Such monolithic category distinctions might profoundly affect decisions and judgements (Monaghan et al., 2012), and separate the experimental data from sound symbolism in natural language, where clear distinctions in sound and meaning are rarely provided. Thus, there is a disjoint between studies purporting to show the relevance of sound symbolism in naturalistic communication, because the constrained sound and meaning spaces are prepared and potentially biased within the mind of the participant.

To show how sound symbolism might infiltrate communication and communicative systems, then, we need data where there is a greater degree of openness over the possible ways in which referents might relate to sound distinctions in language. Such studies require either a large range of potential referents to be presented onto which particular words can be mapped (e.g., Carr et al., 2017), or even less constrainedly, participants could be given free rein to describe the referents to which they feel certain sounds might relate. In an iterative learning study, Kirby et al. (2008) gave participants meanings that varied across shape, colour and movement, and random strings of letters. Over multiple generations, subsets of letters began to systematically reflect the three meaning distinctions. Similar effects were observed by Smith and Wonnacott (2010) for referents that varied over numbering of animals, and Carr et al. (2017) found that words altered from generation to generation of learner to reflect in sound similarities among a set of triangles varying in angles and orientations.

However, these studies are not able to provide insight into how sound distinctions might map onto a completely unconstrained meaning space. Furthermore, these studies tend to indicate how *systematicity* in structure, i.e., statistical regularities between word forms that can predict their meanings, develops within sound-meaning mappings. What these studies do not do is indicate when or how *iconicity,* i.e., aspects of words’ forms that resemble their meanings, emerges in the mappings (Dingemanse et al., 2015). One way to test this is to ask participants to produce meanings given sounds. To our knowledge, only one previous study has tested production of referents given particular words as prompts. Graven and Desebrock (2018) presented the nonwords ‘kiki’ and ‘bouba’ auditorially, and participants drew images of the nonwords. Drawings were judged as more angular for ‘kiki’, and more rounded for ‘bouba’. However, this production task followed a previous task where the words and possible referents were already given to participants that reflected the angular-rounded distinction, likely biasing participants’ drawings.

In this study, we tested whether sound symbolism affected participants’ production of figures based on properties of consonants and vowels in a set of novel words. We predicted that, if sound symbolism can be observed as a driver of construction of individual sound-meaning mappings, then we would see systematic properties of the figures emerge as a consequence of the phonology of the novel words. In particular, we focused on observing iconicity in terms of the angularity or roundedness of figures in response to consonant and vowel properties of the novel words. We distinguished plosive from sonorant consonants, and front versus back vowels in the novel words, and predicted that we would see more straight lines and angular corners for plosives than sonorants and front compared to back vowels. Furthermore, we predicted that consonants in the novel words would drive responses more strongly than vowels, but that both would be influential, as both have been observed in sound-symbolism studies when distinctions in sound and meaning categories are provided to participants (e.g., Monaghan et al., 2012; Nielsen & Rendall, 2012), though vowels would result in a smaller effect (Fort et al., 2015; Ozturk et al., 2013).

As an additional measure, we determined whether consonants and vowels could affect not only the shape but also the complexity of the shapes. It may be that longer lines or more angular corners might reflect not only angularity or roundedness judgements, but also a sense of the complexity of the figures. To determine whether the resulting figures speak to angularity or roundedness and not complexity of shapes, we also measured whether there was a correspondence between the sounds of the novel words and shape complexity. Conceptual complexity has been related to word length (Lewis & Frank, 2016), but not, to our knowledge, to characteristics of figures (Guo et al., 2013).

## Method

### Participants

The study initially tested 42 participants, who were recruited using the Lancaster University Psychology departmental recruitment system as well as through word of mouth. Two of the participants were not included in the analysis because they were unable to produce any figure in response to two of the novel words.

For the remaining 40 participants (33 female, seven male), mean age was 18.6 years (*SD* = 1.5). All participants reported having normal hearing and vision. Thirty-two participants were monolingual English speakers, and eight were bilingual. For those bilingual participants, English was one of their primary languages, with five participants reporting Cantonese as their other language, two reporting Romanian, and one Hindi as their other language.

### Materials

The stimuli comprised 20 novel words formulated by combining either unvoiced plosive or sonorant consonants with either front or back vowels. This resulted in four combinations of consonants and vowels: unvoiced plosive and front vowels, unvoiced plosive and back vowels, sonorant and front vowels, and sonorant and back vowels.

To ensure some generality to the results (i.e., not using just a single example of each nonword), we selected/p/,/t/and/k/for the unvoiced plosives, and the sonorants were /l/, /m/, /n/, /ɹ/, /w/ and /j/. For front vowels, we used /ɛ/ and /i/, and /ɑ/, /ɔ/and /u/ as the back vowels. Note that the front vowels were unrounded, and two of the three back vowels were rounded. In supplementary analyses, we tested whether defining the vowels in terms of their roundedness rather than front or back position resulted in better accounting of the data. We found that it did not (with the exception of predicting complexity ratings where both front/back vowels and roundedness were neither significant). The details of the analyses are reported in Online Supplementary Information S1. The stimuli were constructed from three consonant-vowel syllables (i.e., CVCVCV). The novel words were produced with a British English accent using the Sound of Text website. The list of nonword stimuli are shown in Table 1. Using a variety of phonemes in the stimuli also avoids biasing participants to respond to particular sound distinctions that would be more apparent with a smaller number of stimuli (for instance in previous sound-shape sound symbolism studies that use as few as two stimuli; Ramachandran & Hubbard, 2001).

**Table 1:** List of nonword stimuli classified by consonant and vowel type

Consonants		Plosive	Plosive	Sonorant	Sonorant
Vowels	Front		Back	Front	Back
	pikɛtɛ		pɔkutɔ	nimilɛ	nɔɹulɔ
	kipitɛ		tɔpɔkɑ	lilimɛ	jɑmɔlɑ
	tɛkiki		tɔkupɑ	nɛɹilɛ	wɑlɔmɑ
	tipikɛ		kɑtɔpɔ	mɛjini	mɔlumɔ
	kɛtipɛ		kɑputɔ	milimɛ	lɔnɔwɔ

For collecting drawing responses, we used the Collanote free-line drawing application on a touchscreen tablet. Each participant was provided with a virtual 20-page blank notepad with a set pixel-per-page ratio created on Collanote and given an Apple Pencil to draw the freehand figures.

### Procedure

The study was conducted in-person in a one-on-one session with the experimenter. After giving informed consent, the participant was instructed to draw simple figures that came to mind after listening to each sound by using the given pencil and touchscreen. Participants were asked to attempt each drawing without taking the pencil off the screen. Participants heard each word twice, presented auditorially. If the participant was unable to produce a mental figure at that moment for a word, they were permitted to leave it, and in this case, the participant completed all the other figures and then went back to complete the remaining figures. The participant was gently reminded of the instruction if they repeatedly took the pen off the iPad while drawing the figures. Novel words were presented in randomised order. The entire session took less than 20 min. The participant was debriefed about the aims of the study upon completion and awarded the course credit for their participation.

### Coding and analysis

The figures were manually coded with four measures.

- *Line length:* The length of the straight and the length of the curved lines within each drawing were calculated by tracing over the drawing using ImageJ software (Schneider et al., 2012) with a computer mouse. Straight lines were first traced, then the curved lines in the figures were traced. A curved line was defined as a continuous line with gradual deviation from straight, and a straight line was defined as a continuous line with no deviation from straight.
- *Corner count*: The number of angular and rounded corners used in each figure was counted. A corner was defined as a change in direction of the line. Rounded corners were defined as a smooth change in direction which formed a gradual curve, and angular corners were defined as a sharp directional change which was formed by two lines meeting at a distinct angle.
- *Roundedness-angularity rating*: Each drawing was rated on a 1–10 rating scale (1 = highly rounded and 10 = highly angular).
- *Complexity rating*: Each figure was subjectively rated on a 1–10 rating scale to determine its level of complexity (1 = highly simple and 10 = highly complex).

**Fig. 1:**
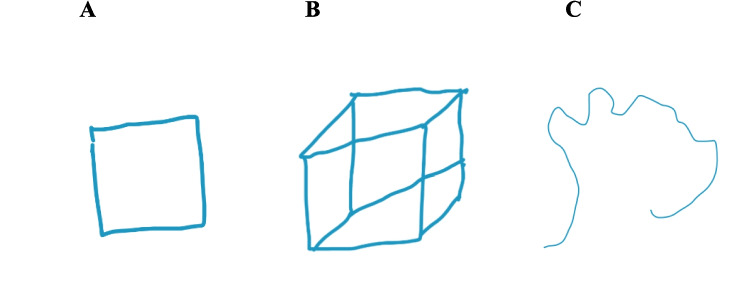
Example of three produced figures. (**A**) Rated high on angularity, low on complexity; (**B**) rated high on angularity, high on complexity; (**C**) rated low on angularity, and medium on complexity

Table 2 reports the measures for each of the three produced figures shown in Fig. 1.

**Table 2:** Measures of straight and rounded line length, number of angular and rounded corners, and angularity and complexity ratings

Measure	A	B	C
Straight Line Length	21.6	106.3	20.6
Curved Line Length	0	0	89.0
Angular Corner Count	4	10	1
Rounded Corner Count	0	0	5
Roundedness/Angularity Rating	9	9	3
Complexity Rating	2	9	6

The inter-rater reliability was calculated by encoding 10% of the figures by a second coder and analysing agreement using Kendall’s *W*. Table 3 shows the agreement, which was high for all measures.

**Table 3:** Kendall’s W for the four coding measures (note that straight and curved lines and corners were assessed for reliability separately)

Parameter	W	χ2(79)	*p*
Straight Line Length	0.860	136	<.001
Curved Line Length	0.877	139	<.001
Angular Corner Count	0.927	147	<.001
Rounded Corner Count	0.938	148	<.001
Roundedness/Angularity	0.859	136	<.001
Complexity	0.807	127	<.001

### Analysis

For each dependent variable, we conducted mixed-effects modelling.

For length of lines, we conducted generalised mixed-effects modelling. The distribution of line lengths in the shapes resembled an inverse Gaussian and so we selected this as the function in mixed-effects modelling of the results, with line length as dependent variable, and random effects of participants and stimuli. We added 0.1 to each line length, as the inverse Gaussian function requires all values to be greater than 0. For participants, we initially included the intercept and consonants (plosives or sonorants), vowels (back or front), line type (curved or rounded), and the interaction between consonants and vowels as random slopes. For stimuli, we included intercepts and line type as slope. We then simplified the random effects structure to the most complex model that was not singular, by removing the participants’ slope for the interaction between consonants and vowels. We then added in fixed effects one by one and tested whether they significantly improved model fit using log-likelihood comparisons. If the fixed effect improved fit, it was retained, otherwise it was not included. We first tested the effect of consonants to determine whether plosives or sonorants affected overall line length. In the same model, we then tested the effect of vowels to see if front or back vowels affected line length production. After these main effects were introduced, we then tested the interaction between consonants and vowels on overall line length to see whether plosives in combination with front or back vowels affected line length production in a different way to sonorants in combination with front or back vowels. Next, we tested the effect of line type, then, crucially, the interaction between line type and consonant, and finally the interaction between line type and vowel. These interactions tested whether length of curved relative to straight lines varied for plosives versus sonorants, and also, in the same statistical analysis, whether the relative lengths of curved and straight lines varied for novel words containing front versus back vowels.

For number of corners as the dependent variable, the distribution matched the Poisson distribution, with random effects constructed in the same way as for line length, and models constructed incrementally by adding each fixed effect and interaction and testing improvement in model fit using log-likelihood tests.

For ratings of the roundedness-angularity and complexity of the figures, we modelled the data using ordinal cumulative link-mixed models, with rating as the dependent variable. Again, random effects were constructed and fixed effects tested in the same way as for the previous analyses.

## Results

Table 4 shows the mean values for each dependent variable for the figures, separated into measures of figures produced in response to novel words containing either plosive consonants with front vowels or back vowels, and sonorant consonants with front or back vowels.

**Table 4:** Mean values for figures produced in response to novel words containing plosive consonants with either front and back vowels, or sonorant consonants with either front or back vowels, for length of straight and curved lines, number of angular and rounded corners, rounded-angularity ratings, and complexity ratings (standard deviations in parentheses)

Consonants	Vowels	Measure					
		Length straight lines	Length curved lines	Number angular corners	Number rounded corners	Round-angular rating	Complexity rating
Plosive	Front	48.02 (24.18)	12.62 (17.26)	5.55 (1.77)	1.16 (0.98)	8.42 (0.88)	5.40 (1.62)
	Back	41.87 (23.60)	25.42 (14.90)	4.28 (1.70)	2.34 (1.19)	6.81 (1.01)	5.29 (1.47)
Sonorant	Front	10.84 (10.59)	50.44 (23.18)	1.45 (1.04)	4.31 (1.45)	3.82 (0.79)	4.84 (1.49)
	Back	1.98 (3.23)	53.65 (22.93)	0.60 (0.48)	4.41 (1.70)	2.64 (0.43)	4.51 (1.17)

### Line length

There was no overall significant effect of consonant on line length (*χ*^*2*^(1) = 2.84, *p* =.092), nor of vowel (*χ*^*2*^(1) = 0.06, *p* =.812). There was a significant interaction between consonant and vowels (*χ*^*2*^(3) = 12.03, *p* =.007). This was due to longer overall lines for back versus front vowels in plosive novel words, *z* = 2.21, *p* =.027, but not in sonorant novel words, *z* = 1.69, *p* =.091, and longer lines for plosives than sonorants in back vowel novel words, *z* = 3.66, *p* <.001, but not in front vowel novel words, *z* = 0.46, *p* =.649.

**Fig. 2:**
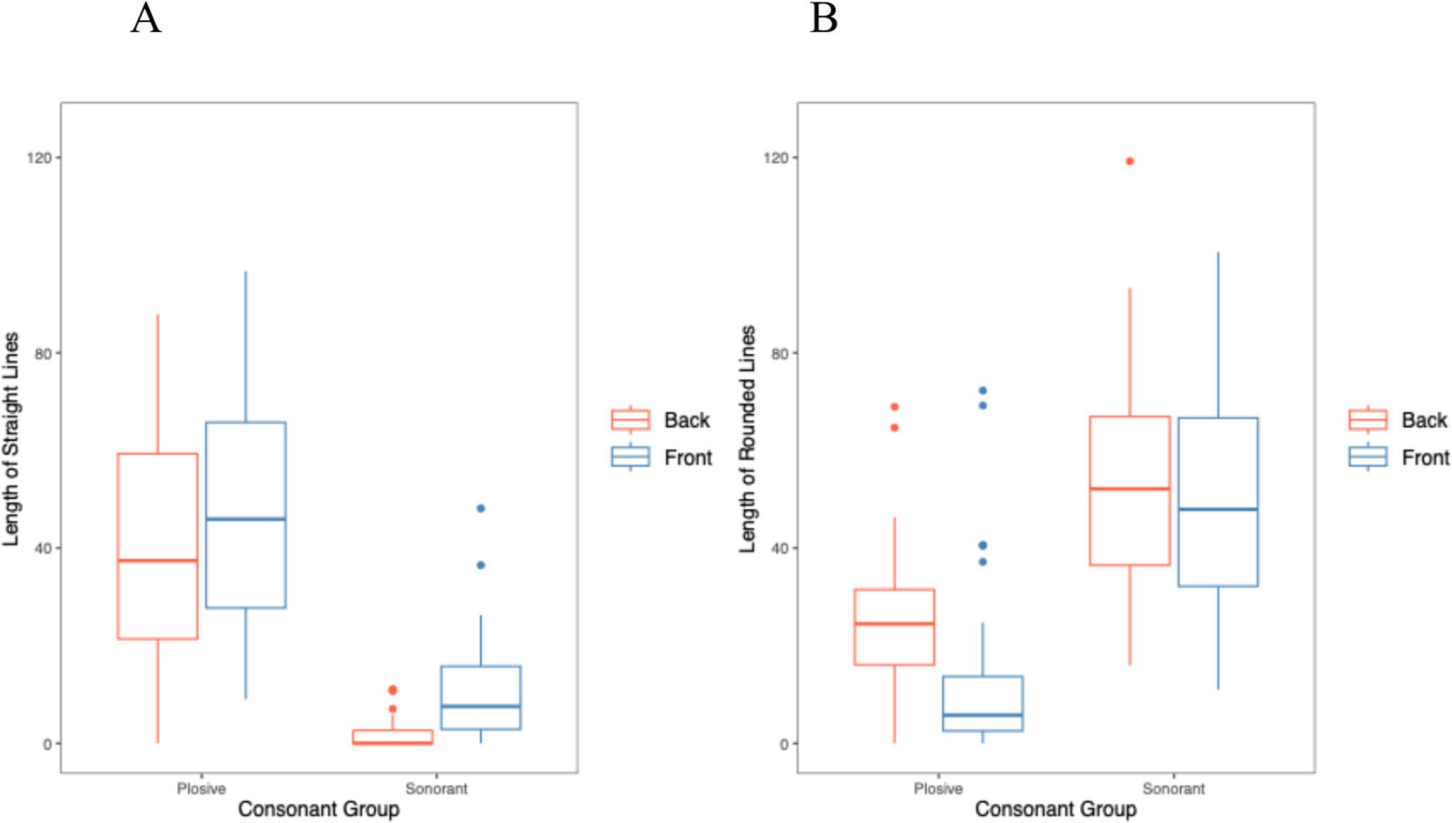
Line length for (**A**) straight and (**B**) curved lines, for stimuli containing consonants and vowels

However, the key results were the effect of the novel words’ properties on length of different line types. There was a significant main effect of line type (*χ*^*2*^(1) = 168.45, *p* <.001), with longer straight lines than curved lines overall. For the interaction between consonants and line type, there was a significant effect (*χ*^*2*^(1) = 759.76, *p* <.001). This was due to longer straight lines for plosives than sonorants, *z* = 11.65, *p* <.001, and longer curved lines for sonorants than plosives, *z* = 14.78, *p* <.001, and also due to longer straight lines than curved lines for plosives, *z* = 14.62, *p* <.001, and longer curved than straight lines for sonorants, *z* = 12.95, *p* <.001, as predicted (see Fig. 2).

For the interaction between vowels and line type, there was also a significant effect in the predicted direction (*χ*^*2*^(1) = 67.09, *p* <.001). This was due to longer straight lines for front than back vowels, *z* = 5.97, *p* <.001, longer curved lines for back than front vowels, *z* = 5.77, *p* <.001, longer curved than straight lines for back vowels, *z* = 10.44, *p* <.001, but no difference between length of straight and curved lines for front vowels, *z* = 0.63, *p* =.529. The interaction between consonants, vowels, and line type was not significant (*χ*^*2*^(1) = 2.64, *p* =.104). Figure 2 shows the effects of consonants and vowels, and line type on line length. Table 5 shows the final mixed-effects model results.

**Table 5:** Final inverse Gaussian mixed-effects model of line length

Predictors	Estimate	Std. error	CI	Statistic	*p-*value
(Intercept)	3.82	0.08	3.66–3.99	46.36	**<0.001**
Line type (curved vs. straight)	0.52	0.06	0.40–0.65	8.20	**<0.001**
consonant (plosive) × vowel	−0.71	0.08	−0.86 – −0.56	−9.14	**<0.001**
Consonant (sonorant) × vowel	0.04	0.05	−0.06 – 0.15	0.85	0.395
Consonant (plosive) × vowel (front)	−1.57	0.14	−1.83 – −1.30	−11.55	**<0.001**
Consonant × line type	−3.11	0.17	−3.44 – −2.78	−18.44	**<0.001**
Vowel × line type	0.97	0.14	0.69–1.24	6.94	**<0.001**
**Random effects**					
σ^2^	460.58				
N _Participant_	40				
N _stimulus_	20				
Observations	1600				
Marginal R^2^/Conditional R^2^	0.001/0.202				

Syntax in R: glmer(linelength+0.1 ~ consonant:vowel + linetype + consonant:linetype + vowel:linetype + (1 + consonant+vowel | Participant) + (1 | stimulus), family = gaussian(link = ‘log’))

### Corners

There was a significant effect of consonant (*χ*^*2*^(1) = 11.26, *p* <.001), with plosives resulting in more corners than sonorants. Vowel was not significant (*χ*^*2*^(1) = 1.96, *p* =.161), and nor was the interaction between consonant and vowels (*χ*^*2*^(2) = 3.97, *p* =.137). There was also no significant main effect of corner type (*χ*^*2*^(1) = 0.932, *p* =.334) with longer straight lines than curved lines overall.

For the interaction between consonants and corner type, there was a significant effect (*χ*^*2*^(1) = 1515.9, *p* <.001) with more angular corners for plosives than sonorants, and more rounded corners for sonorants than plosives, as predicted.

**Fig. 3:**
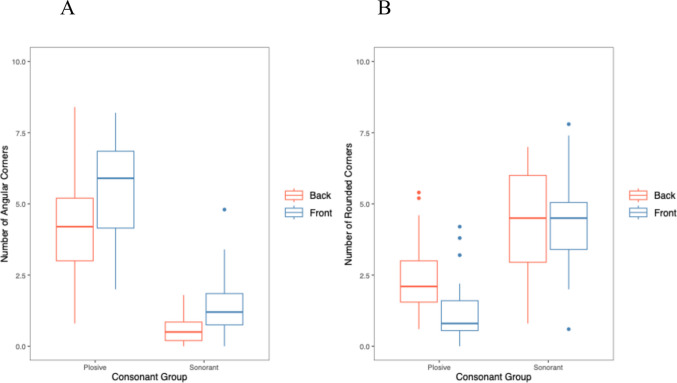
Number of corners for (**A**) angular and (**B**) rounded corners, for stimuli containing consonants and vowels

For the interaction between vowels and corner type, there was also a significant effect (*χ*^*2*^(1) = 157.21, *p* <.001) with more angular corners for front than back vowels, and more rounded corners for back than front vowels. The interaction between consonants, vowels and corner type was also significant (*χ*^*2*^(2) = 24.656, *p* <.001), showing a larger effect of consonants and vowels for angular corners than for rounded corners. Figure 3 shows the effects of consonants and vowels, on number of each corner type; see Table 6 for final model.

**Table 6:** Poisson mixed-effects model of number of corners

Predictors	Estimate	Std. error	CI	Statistic	*p*-value
(Intercept)	4.16	0.26	3.69–4.70	23.26	**<0.001**
Consonant	0.14	0.02	0.11–0.17	−17.34	**<0.001**
Consonant [P] × corner type	0.55	0.03	0.49–0.61	−10.54	**<0.001**
Consonant [S] × corner type	7.40	0.72	6.12–8.96	20.58	**<0.001**
Corner type [angular] × vowel	1.29	0.09	1.13–1.48	3.64	**<0.001**
Corner type [round] × vowel	0.49	0.05	0.41–0.60	−7.32	**<0.001**
Consonant [S] × corner type [angular] × vowel	1.89	0.26	1.44–2.48	4.63	**<0.001**
Consonant [S] × corner type [round] × vowel	1.99	0.23	1.58–2.51	5.85	**<0.001**
**Random effects**					
σ^2^	0.29				
N _Participant_	40				
N _stimulus_	20				
Observations	1600				
Marginal R^2^/Conditional R^2^	0.613/0.687				

Syntax in R: glmer(corners ~ consonant + consonant:cornertype + vowel:cornertype + consonant:vowel:cornertype + (1 + consonant+vowel | Participant) + (1| stimulus), family = poisson(link = ‘log’))

### Roundedness-angularity rating

**Fig. 4:**
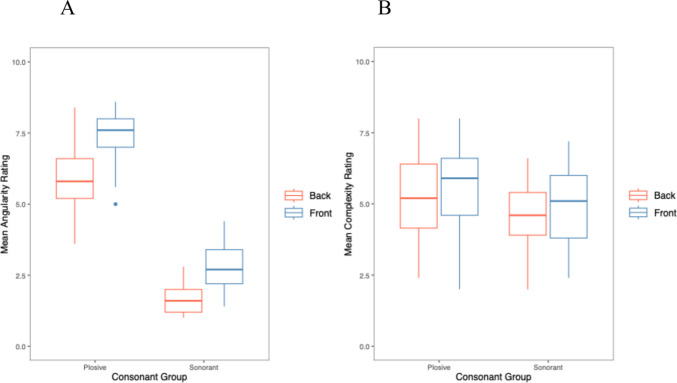
Ratings for (**A**) roundedness-angularity and (**B**) complexity, for stimuli containing consonants and vowels

There was a significant effect of consonant (*χ*^*2*^(1) = 41.13, *p* <.001), with plosives resulting in higher angular ratings than sonorants. Vowel was also significant (*χ*^*2*^(1) = 60.80, *p* <.001), with higher angular ratings for front than back vowels. There was no significant interaction between consonant and vowels (*χ*^*2*^(1) = 0.28, *p* =.594). Figure 4A shows the effects of consonants and vowels on mean ratings; Table 7 shows the final model.

**Table 7:** Cumulative link for mixed-model results for roundedness-angularity ratings

Predictors	Estimate	Std. error	CI	Statistic	*p*-value
1|2	0.00	0.00	0.00–0.00	−10.74	**<0.001**
2|3	0.00	0.00	0.00–0.01	−19.76	**<0.001**
3|4	0.03	0.01	0.02–0.05	−13.99	**<0.001**
4|5	0.12	0.03	0.08–0.19	−9.80	**<0.001**
5|6	0.28	0.05	0.19–0.40	−6.75	**<0.001**
6|7	0.67	0.11	0.48–0.94	−2.34	**0.019**
7|8	1.67	0.28	1.20–2.32	3.07	**0.002**
8|9	5.49	0.99	3.86–7.82	9.47	**<0.001**
9|10	30.09	6.84	19.27–46.97	14.98	**<0.001**
10|11	2423.53	2480.15	326.10–18011.03	7.62	**<0.001**
Consonant	0.00	0.00	0.00–0.01	−19.67	**<0.001**
Vowel	8.08	1.29	5.91–11.05	13.08	**<0.001**
**Random effects**					
σ^2^	3.29				
N _Participant_	40				
N _stimulus_	20				
Observations	800				
Marginal R^2^/Conditional R^2^	0.729/NA				

Syntax in R: clmm(rating_angular ~ consonant + vowel + (1 + consonant*vowel | Participant) + (1 | stimulus))

### Complexity ratings

There was a significant effect of consonant (*χ*^*2*^(1) = 10.25, *p* <.001), with plosives resulting in higher complexity ratings than sonorants. There was no significant effect of vowel (*χ*^*2*^(1) = 2.29, *p* =.131), and no significant interaction between consonant and vowels (*χ*^*2*^(1) = 0.28, *p* =.594). Figure 4B illustrates the mean ratings by consonants and vowel groups, and Table 8 reports the final model.

**Table 8:** Cumulative link mixed-model results for roundedness-angularity ratings

Predictors	Estimate	Std. error	CI	Statistic	*p*-value
1|2	0.01	0.00	0.00–0.01	−14.16	**<0.001**
2|3	0.18	0.04	0.12–0.28	−8.17	**<0.001**
3|4	0.33	0.07	0.22–0.48	−5.56	**<0.001**
4|5	0.51	0.10	0.34–0.75	−3.41	**0.001**
5|6	1.00	0.20	0.68–1.48	0.01	0.989
6|7	2.30	0.46	1.55–3.42	4.14	**<0.001**
7|8	5.74	1.21	3.79–8.68	8.27	**<0.001**
8|9	22.42	5.57	13.78–36.48	12.53	**<0.001**
Consonant	0.57	0.09	0.41–0.78	−3.43	**0.001**
**Random effects**					
σ^2^	3.29				
N _Participant_	40				
N _stimulus_	20				
Observations	800				
Marginal R^2^/Conditional R^2^	0.024/NA				

Syntax in R: clmm(rating_complexity ~ consonant + (1 + consonant*vowel | Participant) + (1 | stimulus))

## Discussion

Our results showed that the characteristics of participants’ drawings are strongly affected by the sounds of the novel words that they hear. Without constraining, or cueing, participants to any dimensions of meaning, we found that participants produced more straight lines than curved lines for plosives and front vowels, and more curved lines than straight lines for sonorants and back vowels. Similarly, plosives and front vowels drove production of figures with more angular than rounded corners, whereas sonorants and back vowels resulted in figures with more rounded than angular corners. When these figures were rated for their angularity or roundedness, the ratings were again consistent with these distinctions in consonant manner and vowel place features. Complexity ratings were found to be less consistently related to both consonant and vowel properties, though we had not made predictions about these relations as they were included as a control measure for the structure of the figures. Complexity is known to relate to word length, rather than particular phonological properties of words (Lewis & Frank, 2016). The results reflect this control property: the complexity ratings of the figures did not relate significantly to the sound properties of the novel words that stimulated the figures. Thus, the key differences associated with speech sounds are related to the shape, rather than the detail, of these shapes.

The results are thus consistent with the classic bouba-kiki or takete-maluma effect (Ramachandran & Hubbard, 2001; Köhler, 1929; Nielsen & Rendall, 2012; Sidhu et al., 2021): both consonants and vowels affect the creation of shapes that relate to angularity and roundedness. Thus, when participants are given free reign to produce figures in response to these speech sounds, and when they are not cued in any way to how these figures should be generated, they nonetheless show strong tendencies to reproduce distinctions in shape that mimic the standard laboratory-based studies on this sound-shape correspondences. This shows that the bouba-kiki effect is not limited to studies that provide both the sound and the meaning distinctions, requiring forced choices in the mappings, and show that, without constraints on participants’ productions, other than that they should draw a figure without taking their pen from the surface, they preferentially generate referents that reflect sound symbolism.

How do these results relate to natural language structure and processing? There is some evidence that the angular-rounded distinction is reflected in speech properties. Monaghan et al. (2012) conducted an analysis of 509 expressives for roundedness or angularity in English, and found a small but statistically significant correspondence between voicing and the angular/rounded distinction. Sidhu et al. (2021) asked participants to provide spiky and rounded judgements for 1,757 objects, and found that the words in English used to name these objects showed an association between /k/, /t/and /i/ and spiky objects, and /u/, /m/ and /b/ and rounded objects, amongst other phonemes. Thus, the classic bouba-kiki or takete-maluma effect has been found in at least one natural language, i.e., English (Sidhu, 2025).

It may be that natural language drives the construction of angular and rounded shapes from consonant and vowel properties of novel words in our study. The small biases found in English words might be sufficient to influence the shapes in our study. Styles and Gawne (2017), for instance, showed that in a forced-choice task, when there was a mismatch between the phonotactics of the natural language and the experimental stimuli (i.e., the stimuli did not resemble the sound patterns found in the natural language), then null effects for maluma/takete effects tend to follow. Thus, natural language experience has a vital role in driving experimental results.

However, our results seem to go beyond the regularities found in the natural language of our participants. Several of the phonemes used in our study were not found to be statistically related to angularity or roundedness in English. For vowels, /u/, /ɔ/ and /i/ were consistent between our study results and Sidhu et al.’s (2021) study, yet we also included the back vowel/ɑ/and the front vowel /ɛ/, which were not found to be consistently related to the meaning distinction in English. For consonants,/k/,/t/and/m/were consistent between our study results and Sidhu et al. (2021), but /p/, /l/, /j/, /w/ and /n/ were not, though the voiced-unvoiced distinction found in Monaghan et al. (2012) for rounded and angular expressives was consistent with our consonant materials. Online Supplementary Information S2 reports the results for each phoneme individually, and the results are remarkably consistent across the different phonemes within the plosive/sonorant distinction and front/back vowel distinction. Thus, the effects we observed are not constrained only to the sound-meaning mappings found in the statistics of natural language.

An alternative account is that there is a common origin of the effect that results in the presence of the sound symbolism in (small pockets) of natural language, and in experimental study results. One possibility is that the correspondence may result from environmental associations among sensory stimuli (Sidhu & Pexman, 2017). These may operate within the language: such that there are statistical associations between sound and meaning within the natural language environment – as suggested above – but this does not account for the entire effect observed in the current study, where more general properties of speech sounds (voicing, manner of consonants, and front/back vowels) rather than observed phoneme-meaning correspondences between sound and meaning in English seemed to drive the effects.

Alternatively, the correspondences may be between broader sound and meaning similarities, such as the link between pitch and size – larger objects tend to emit lower pitch (Walker et al., 2012) – which can then be acquired through experience, or encoded in evolved sensitivity to associations. Westbury et al. (2018) noted that sound-meaning correspondences are not independent of one another, such that phonemes relating to roundedness were also those found to relate to large, and phonemes relating to angularity were inversely related to large. Gallace et al. (2011) found that words associated with roundedness also related to tenseness (vs. relaxedness) and high (vs. low) activity (see also Lyman, 1979). Thus, the associations among multiple meanings, which individually contribute correspondences between sound and meaning found in the natural environment, may well be vital to understanding the role of sound symbolism in language processing, and, ultimately, language evolution (Fort & Schwartz, 2022).

## Supplementary Information

Below is the link to the electronic supplementary material.

Supplementary file1 (DOCX 205 KB)

## Data Availability

The materials are outlined in the *Materials* section of this paper. The anonymised data and analysis scripts can be found at https://osf.io/8uvkm/overview?view_only=1dbf68acca844230b6c97847af62f8e4
