# Supplementary material for: The shape of a kiki: Sound symbolism affects production of figures
Source: Psychon Bull Rev. 2026 Feb 17;33(3):72. doi: 10.3758/s13423-026-02864-0 (PMC12913298; doi:10.3758/s13423-026-02864-0)
Supplement: Supplementary file 1 — Supplementary file1 (DOCX 205 KB) [file 13423_2026_2864_MOESM1_ESM.docx]

The shape of a kiki: Sound symbolism affects production of figures

Supplementary Information

**S1. Comparisons between analyses based on front/back vowel and vowel roundedness distinction.**

We measured the proportion of rounded vowels in each stimulus in the study, and entered this as a fixed effect in place of the front/back vowel distinction in the best fitting models from the main paper. We then compared model fit in terms of AIC, BIC, and log-likelihood. Values closer to zero indicate better fit.

***Line length***

|  | AIC | BIC | Log-likelihood |
| --- | --- | --- | --- |
| Front/back | 15493 | 15574 | -7731.4 |
| Rounded | 15571 | 15646 | -7771.6 |

For the interaction between line type and front/back vowel, the results in the final model (Table S1.1) show estimate = 0.97, CI = 0.69-1.24, p < .001. For the interaction between line type and roundedness, estimate = -0.98, CI = -1.27- -0.69, p < .001.

***Corners***

|  | AIC | BIC | Log-likelihood |
| --- | --- | --- | --- |
| Front/back | 6945.4 | 7015.3 | -3459.7 |
| Rounded | 6956.6 | 7026.6 | -3465.3 |

For the interaction between corner type and front/back vowel, the results in the final model (Table S1.2) show estimate = 0.72, CI = 0.60 - 0.86, p < .001. For the interaction between corner type and roundedness, estimate = 1.41, CI = 1.14 - 1.75, p = .002.

***Angularity-roundedness rating***

|  | AIC | Log-likelihood |
| --- | --- | --- |
| Front/back | 2540.6 | -1247.3 |
| Rounded | 2564.9 | -1259.4 |

For the main effect of front/back vowel, the results in the final model (Table S1.3) show odds ratio = 8.08, CI = 5.91-11.05, p < .001. For the main effect of roundedness, estimate = 0.09, CI = 0.06-0.15, p < .001.

***Complexity rating***

|  | AIC | Log-likelihood |
| --- | --- | --- |
| Front/back | 3181.2 | -1569.6 |
| Rounded | 3176.3 | -1567.2 |

For the main effect of front/back vowel, there was no significant main effect in the final model (Table S1.4), but adding front/back vowel to this final model resulted in odds ratio = 1,23, CI = 0.94-1.60, p = .129. For the main effect of roundedness, estimate = 0.81, CI = 0.58 – 1.13, p = .209.

**S2. Descriptive statistics for individual phonemes**

We analysed the first consonant and first vowel of each word separately.

***Line length***

******

***Number of corners***

***Angularity-roundedness rating***

***Complexity rating***

******

******
